# The Impact of Visual and Cognitive Dual-Task Demands on Traffic Perception During Road Crossing of Older and Younger Pedestrians

**DOI:** 10.3389/fpsyg.2022.775165

**Published:** 2022-02-17

**Authors:** Rebecca Wiczorek, Janna Protzak

**Affiliations:** Department of Psychology and Ergonomics, Junior Research Group FANS, Technische Universität Berlin, Berlin, Germany

**Keywords:** older pedestrians, road crossing behavior, dual-task, traffic perception, visual attention

## Abstract

With the help of the current experiment, we wanted to learn more about the impact of visually demanding vs. cognitively demanding secondary tasks on the attention allocation of older pedestrians during the phase of traffic perception within the process of road crossing. For this purpose, we used two different road crossing tasks as well as two different secondary tasks. The road crossing “stop task” was a signal detection task, where an approaching car had to be detected. The road crossing “go task” was a dynamic visual search task, where the resolution of a busy road situation had to be identified. The visual secondary task was a static visual search task and the cognitive secondary task was a 1-back (memory) task. One younger group (≤ 30 years) and one older group (≥ 65 years) of participants completed the tasks as single vs. dual-tasks in all possible combinations. Performance was measured through errors and response time; in addition, the subjective workload was assessed *via* NASA-TLX. Analyses show that the visual secondary task reduces performance in the road crossing more strongly than the cognitive task, while the visual task itself is less impaired by the road crossing tasks than is the cognitive task. Overall, performance diminishes from single to dual-task completion. Results further indicate age effects in terms of increased errors and response time for older compared to younger participants. In addition to these age effects, age-specific dual-task effects emerge for response time in the go task along with the visual task as well as for response time in the cognitive task along with the go task. Subjective workload is higher in the dual-task conditions than in the single tasks. Findings are discussed with regard to theoretical and practical implications.

## Introduction

Walking supports both physical and psychological health (Cirkel and Juchelka, 2007). Therefore, it is a key factor for self-determent living and social participation (Limbourg and Matern, 2009; Hefter and Götz, 2013). Its relevance increases with older age, as more and more everyday tasks, such as shopping for groceries or visiting the doctor, are carried out on foot (Follmer et al., 2010). In the meantime, the use of other transportation methods, such as car, bicycle, or public transportation, diminishes (Follmer et al., 2010). However, with increasing age, the risk of also getting injured as pedestrians in a car accident increases (Rytz, 2006).

Age-related declines of sensory, cognitive, and motoric functions are the underlying reasons for many of these accidents (*cf*. Oxley et al., 2004). However, it is not the decline of abilities *per se* that causes the crashes. It is rather an interplay of certain elements of the complex task of road crossing with specific impairments of older people. According to Older and Grayson (1974), the process of road crossing can be divided into five phases. The existing broad body of research regarding older pedestrians has not focused on all of these phases with the same intensity yet. Only a few studies exist regarding phase one “selection of crossing location” (i.e., Holland and Hill, 2007; Bernhoft and Carstensen, 2008) and phase two “traffic perception” (i.e., Snowden and Kavanagh, 2006; Tapiro et al., 2016; Stafford et al., 2019). Most of the studies regard a combination of phase three and four “traffic analysis” and “crossing-decision” (i.e., Oxley et al., 1997; Holland and Hill, 2010; Dommes and Cavallo, 2011; Lobjois et al., 2013; Naser et al., 2017) or focus on phase five “actual crossing” (i.e., Carmeli et al., 2000; Oxley et al., 2005; Bollard and Fleming, 2013; Dommes et al., 2014; Butler et al., 2016; Geraghty et al., 2016; Duim et al., 2017). For an overview, see the systematic review of Wilmut and Purcell (2021).

However, police statistics of Berlin indicate that the most prevalent (~60%) pedestrian-related reason for older pedestrians becoming the victim of a car accident is the lack of attention toward the ongoing traffic (Statistisches Amt Berlin Brandenburg, 2020). Thus, the intention of the current research was to focus on phase two “traffic perception” investigating why older pedestrians do not look for cars sufficiently when crossing a road.

### Older Pedestrians Lack of Attention Toward Traffic

With the help of an earlier observation study and a group discussion, we could identify potential reasons why older pedestrians sometimes overlook upcoming cars (Wiczorek et al., 2016). One important reason seems to be the engagement in concurrent visual tasks, namely, scanning the ground for obstacles. To further analyze this finding in a next step, a photo-based questionnaire was given to a group of younger and a group of older pedestrians. They were shown different crossing scenarios at various streets and were then asked about their behavior with regard to scanning the ground in case of uneven floors and high curb stones. The results of this quantitative method confirmed findings from the qualitative group discussion and the observation study. Older people indicated significantly more often to check the floor for obstacles compared to younger pedestrians (Wiczorek et al., 2016).

This finding based on self-reports is in line with findings of an observation study by Avineri et al. (2012). They found a positive correlation between checking the ground and established fear of falling, which is more pronounced in older than younger people (Tinetti et al., 1994; Schott, 2008). It is further in line with a laboratory experiment using head tracking by Zito et al. (2015). They compared head and eye movements of younger and older people in a virtual reality road crossing scenario. Even though there were no obstacles on the floor, older people looked down at their feet (instead of looking at the street) significantly more often than younger. Additionally, Tapiro et al. (2016) found older participants to focus longer on the planned walking path (instead of on the traffic) compared to younger participants.

After identifying this behavior as being typical for older pedestrians, we wanted to investigate its potential impact on road crossing performance within a laboratory experiment. Thus, the current study investigates the impact of a visual secondary task on visual attention in two different road crossing scenarios with older and younger people. The visual secondary task was additionally contrasted to a cognitive secondary task, which was integrated in this experiment to learn more about the specific resources requested by different road crossing tasks. Before we go into detail, we would like to give an overview about the state of the art in dual-task research regarding older pedestrians’ performance in road traffic.

### Dual-Task Research Regarding Older Pedestrians in Road Traffic

The impact of dual-task requirements on road crossing of older pedestrians has not been studied excessively, but there are some interesting studies thus far. Neider et al. (2011) compared older people (between 59 and 81 years) with college students, while crossing a road in a pedestrian simulator on a treadmill, varying dual-task requirements. They either crossed the street without an additional task, with the task of listening to music, or with the task of talking at the phone to a real person asking questions. Furthermore, gap lengths between cars served as an additional factor. When gaps were small, road crossing performance of older people decreased when parallel talking on the phone. All the other conditions did not reveal dual-task costs. However, a similar study conducted only with younger subjects could show a negative effect of listing to music while crossing a road (Schwebel et al., 2012). This might have been the case as there was auditory traffic information, which was not present in the study of Neider et al. (2011).

Nagamatsu et al. (2011) used the same paradigm and task as Neider et al. (2011) and divided their group of older participants (above the age of 65) into two groups based on their scores in a risk of falling questionnaire. While both groups’ crossing showed decreased performance in the dual-task with the phone call, the group with higher fall risk performed significantly worse than the one with the lower risk.

Butler et al. (2016) investigated older people’s (aged between 70 and 90 years) road crossing performance with and without an additional task on a simulated road using a mock-up car made from Styrofoam and aluminum. The additional task consisted of putting balls of one color from a jar with balls of two different colors in another jar. When facing the additional task, they turned their back toward the street. Instructions regarding road crossing were to cross as close in front of the car as possible while still being safe. In the single-task conditions, all the subjects crossed the street safely. When being engaged in the additional task, 24% of the subjects started their crossing significantly later than in the single-task condition and had either to interrupt their attempt or got hit by the car. Further perceptual, physical, and cognitive tests indicated a positive relation of these functions with the presented road crossing performance.

Dommes (2019) compared the behavior of younger participants in street-crossing dual-task situations in a pedestrian simulator with the performance of younger-old participants (between 60 and 72 years old) and older-old participants (between 73 and 82 years old). The single task of crossing the street without traffic was compared to two different dual-task crossing situations. One was crossing the street with a signal-reaction task (visual and auditory), and the other one was crossing the street with traffic. All groups walked faster in the single-task condition compared to the dual-task conditions. However, while the younger group walked identically slow in both dual-task conditions, the older groups walked faster in the crossing with traffic condition than in the crossing with signal-reaction task condition. As they prioritized fast walking over scanning for traffic, they were hit by cars significantly more often than younger participants.

The studies described here investigated the impact of different secondary tasks on traffic analysis, crossing decision and actual crossing (road crossing phase three to five, Older and Grayson, 1974), while the current study focusses on phase two “traffic perception.” They all found certain reductions of older pedestrians’ road crossing performance in different dual-task situations. However, there are some differences regarding interference of different combinations of secondary and road crossing tasks. For example, Schwebel et al. (2012) and Neider et al. (2011) had different results regarding the interference of road crossing with the secondary task of “listening to music.” According to their plausible interpretation, this was due to the different nature of road crossing task that either involved or did not involve auditory information. While findings generally point in the same direction, it is not possible to explain why certain secondary tasks interfere with certain road crossing tasks based on recent findings. On reason is the use of close to naturalistic secondary tasks that makes understanding of required resources difficult. The other reason is that most studies keep the road crossing task constant and only vary the secondary tasks. Thus, the impact of differences in road crossing tasks on the interference cannot be assessed.

### Current Study

Within the current study, we aim to learn more about the nature of different road crossing task demands to understand why they interfere (stronger) with different secondary tasks. For this purpose, we designed two different road crossing tasks as well as two different secondary tasks. The road crossing tasks were kept as realistic as possible, while the secondary tasks were rather artificial to increase internal validity regarding the required resources.

According to the Multiple Resource Model (MRM; Wickens, 2002), tasks incorporate three stages: perception, cognition, and response. Perception can use two different modalities (visual and auditory), and response can use two different modalities (manual and verbal). Two tasks interfere with each other when requiring the same resource and when the overall sum of required resource exceeds the available amount. Dual-task costs manifest in reduced performance as a result of parallel completion and can be found in one or in both tasks (i.e., Norman and Bobrow, 1975). A special type of this cost is the age-specific dual-task cost, where the increase in cost from single to dual-task is more pronounced in older than in younger subjects (i.e., Riby et al., 2004).

The two road crossing tasks resemble different everyday tasks. The one task consists of seeing a car and indicating to stop walking (stop task). The other one consists in understanding that a busy traffic situation has resolved and indicating to initiate walking (go task). Both tasks require mainly visual perceptual resources but involve of course cognitive resources for the decision making. The stop task can be characterized as signal detection task, which requires very little capacity of the working memory. The go task instead resembles a dynamic visual search task. With several cars involved it requires more capacity of the working memory as well as the ability for inhibition, that is often impaired in older age (i.e., Tipper, 1991). As we want to focus on phase two “traffic perception” (*cf*. Older and Grayson, 1974), we reduced the motoric demands of the tasks to a minimum. Instead of initiating a whole body movement, participants have to indicate their decision in the road crossing task by pulling or pushing a joystick. Reduction of physical requirements is important as we know form dual-task basic research that older people tend to give priority to motoric tasks over cognitive tasks (posture first effect, Schäfer, 2014).

The secondary tasks were designed in order to create interference with the different stages (perception and cognition) of the road crossing tasks. The original study further included a motoric other task. Due to the very different nature of task and corresponding hypothesis, we published the results of the respective analyses elsewhere (Protzak and Wiczorek, 2017; Siegmann et al., 2017). The one secondary task (visual task) demands resources mainly in the stage of perception. It is a visual search task that requires enhanced visual attention. The other secondary task (cognitive task) demands resources mainly in the stage of cognition. It is a 1-back memory task that is given auditory and requires no visual perceptual resources. The two secondary tasks include no manual action. Instead, we chose the verbal response modality for both secondary tasks.

Table 1 compares the modalities and required amounts of resources of the road crossing and the secondary tasks. Tasks should interfere when using the same modality and when the required amount of resource per stage exceeds the available amount.

**Table 1:** Modalities and amount of required resources for the road crossing and the secondary tasks.

Task	Perception stage	Cognition stage	Response stage
Stop task	High visual requirement	Low cognitive requirement	Low motor requirement
Go task	High visual requirement	Medium cognitive requirement	Low motor requirement
Visual task	High visual requirement	Low cognitive requirement	Low verbal requirement
Cognitive task	Medium auditory requirement	High cognitive requirement	Low verbal requirement

Colors indicate modalities and color intensity indicates amount of required resource (Stronger) interference is expected for same colors with medium to high intensities.

We do not frame the experiment with different priorities for the two tasks. Instead, participants are told that both tasks are equally important. This instruction is given to create a competitive situation between the two tasks that resemble reality. Prioritization of tasks is part of the dual-task requirements and allows us to investigate whether the different combinations of road crossing tasks and secondary tasks trigger different behavioral strategies.

Within the current study, we want to investigate if and how different secondary tasks interfere with the phase of “traffic perception” in two different road crossing tasks. We expect the visual task to interfere more with the road crossing tasks than the cognitive task. Furthermore, we expect an age-specific dual-task effect. Performance decrements from single to dual-task conditions should be greater for older compared to younger participants.

## Materials and Methods

“Ethik-Kommission des Instituts für Psychologie und Arbeitswissenschaft (IPA) der TU Berlin” approved the study under the name: “Laborstudie zum Verhalten im Straßenverkehr” (serial numbers SIE_01_20160329). All procedures were performed in accordance with the Declaration of Helsinki, in compliance with relevant laws and institutional guidelines. Written informed consent was obtained from each participant and privacy rights were observed.

### Participants

Thirty-eight participants were recruited for the study. Half of them was labeled “younger,” and ranged from age 18 to 30 (*M* = 25.58; *SD* = 3.56), six of them were male, 13 were female. The other group of participants was labeled “older,” and ranged from age 67 to 82 (*M* = 71.16; *SD* = 3.73), six of them were male, 13 were female. All participants completed a test of visual ability (younger: *M* = 99%; *SD* = 22%; older: *M* = 63%; *SD* = 15%) and the Montreal Cognitive Assessment (MoCA, Nasreddine et al., 2005; younger: *M* = 27.53; *SD* = 1.92; *M* = 25.84; *SD* = 2.77). All participants of both groups stated to walk on a regular basis. The *younger* participants were recruited from the participants data base of the “Institute of Psychology” of the “Technische Universität Berlin” that contains mainly students, while the *older* participants were recruited from a participants data base of the research group “FANS” of the “Technische Universität Berlin” that contains people with an age between 60 and 90 years. Younger and older subjects received a participation compensation of 10€ and 12€ per hour, respectively.

### Task Environment and Apparatus

The experiment took place in the pedestrian simulation laboratory of the FANS research group at the Technische Universität Berlin. Participants wore a headset and were standing at a standing desk equipped with a joystick. In front of them was a 1.5× 5 m (high × wide) projection of a street environment (3,810 × 1,080 pixel), displayed by two projectors (Acer S1283 HNE). Videos of street scenes for the road crossing tasks were built with the open-source software Blender. Road crossing and other tasks were presented using the python-based open-source software package PsychoPy (Peirce, 2007).

**The two road crossing tasks** consist of short videos of street scenes. In total, we have six different street scenes, three for each road crossing task. The stimuli are crossing cars, while some of the videos also contain distractors in the shape of e-bikes driving in the opposite sidewalk. In each experimental block, participants conduct either the stop task or the go task. A block consists of 15 videos à 20s, five videos of each street scene. While the order of streets is counterbalanced, the five street scenes of the same street are always presented in a row to make prediction of events more difficult.

**Figure 1:**
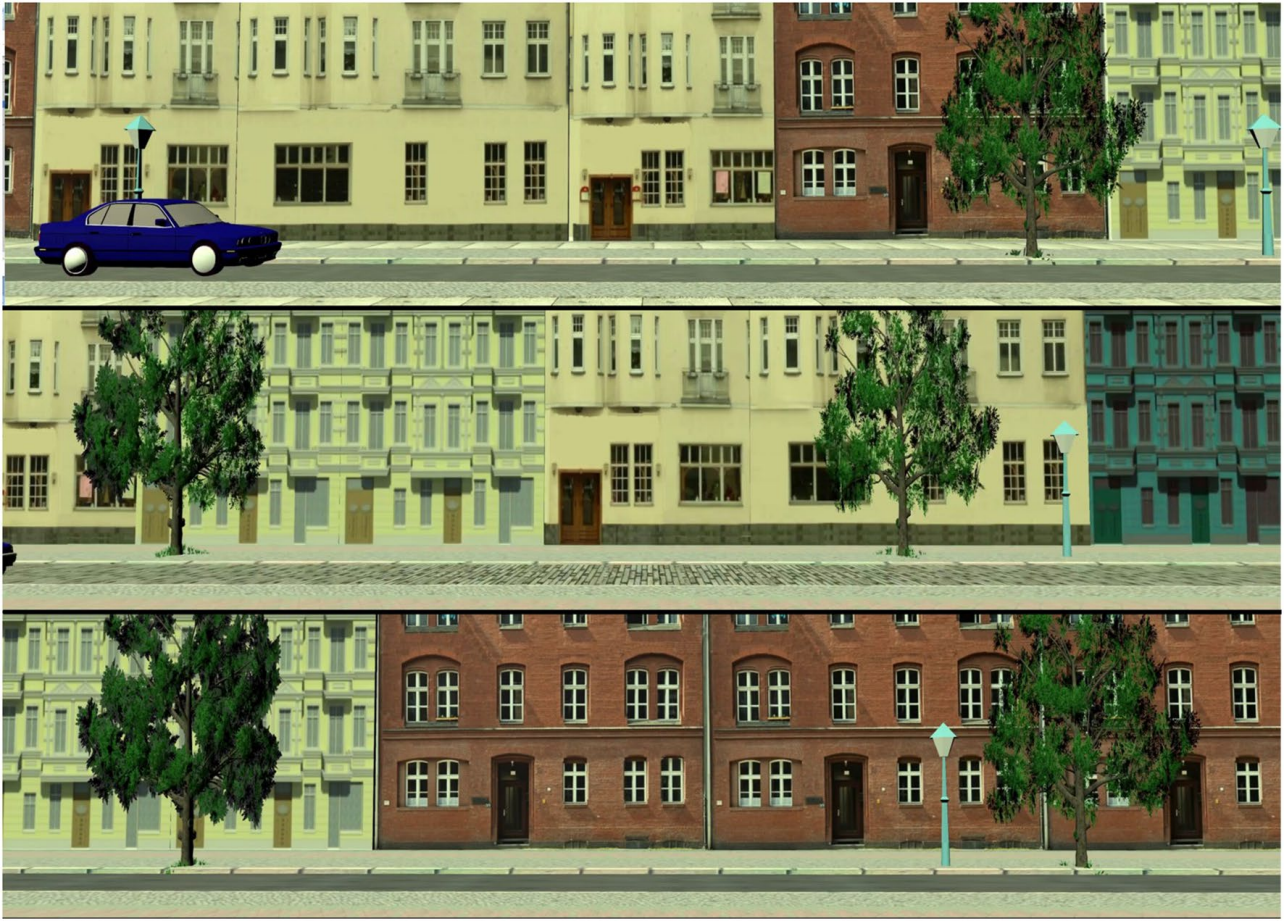
The three different street scenarios of the stop task, with one car approaching from the left side in the first scenario.

**In the road crossing stop task,** participants see an empty road. At a non-predictable moment, a car crosses the street from the left or from the right side. Participants’ task is to pull the joystick when they see a car, indicating they would stop. They are instructed to respond as fast and as correct as possible. Non-responding to a car is counted as an error as well as pulling the joystick in the absence of a car (for example, as a reaction to an e-bike). The stop task is a signal detection task. Figure 1 shows the three street scenarios of the stop task.

**In the road crossing go task,** participants see a busy road with cars crossing form the left and the right side. At a non-predictable moment, no new cars appear and the road turns empty (and stays empty for the rest of the scene). Participants’ task is to push the joystick, indicating they would “go,” i.e., start crossing the street. They are instructed to respond as fast and correct as possible. Non-responding is counted as an error as well as responding to early (i.e., before the street is finally empty).

**Figure 2:**
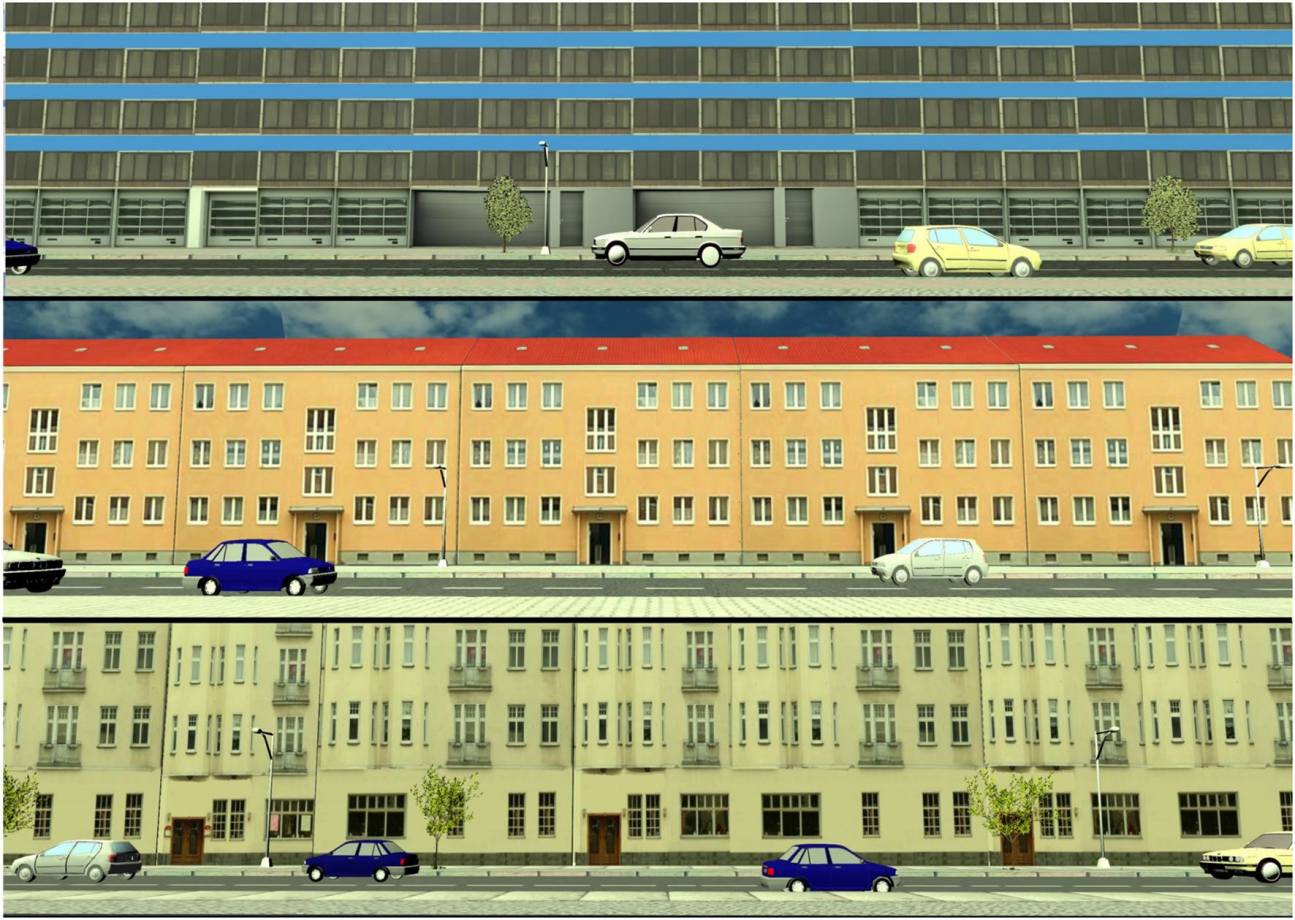
The three different street scenarios of the go task, with cars crossing from both sides.

This task is similar to often used gap selection tasks (e.g., Oxley et al., 2005). However, there are important differences with regard to the underlying abilities required for the task. The main focus of a gap acceptance task is to decide whether an approaching car is far enough away and slow enough to allow a crossing. Thus, this task challenges the ability to correctly judge speed and distance, and, to integrate these two pieces of information to make a decision. Instead, the main focus of the present go task is to quickly notice when a busy situation is resolving. Hence, the required ability is the inhibition of distractors, which is often found to be diminished in older people (i.e., Tipper, 1991). Therefore, the go task is a sort of dynamic visual search task. Figure 2 shows the three street scenarios of the go task.

**Figure 3:**
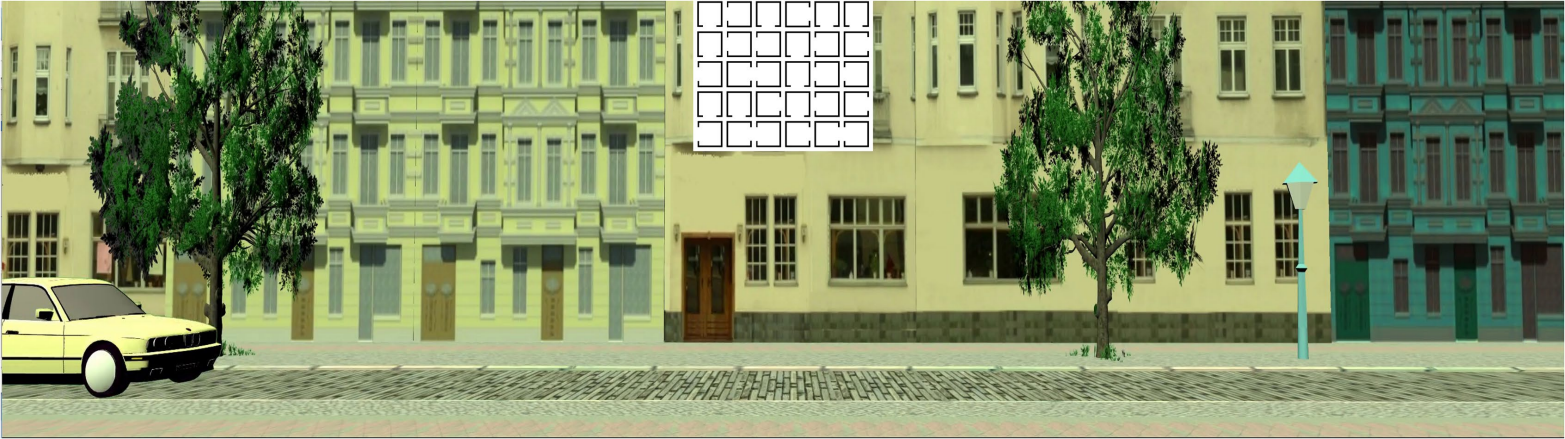
The visual task along with the stop task, with one car approaching from the left side.

**The visual secondary task** is displayed in the upper middle part of the scene. It consists of a 5×6 matrix of white squares on black ground (or vice versa, color type is alternating to indicate changes of stimuli). Squares are open at one of the four sides. Each matrix either contains one or zero squares that are open at the top side. Participants respond “yes” (for matrices with squares that are open at the top) or “no” (for matrices with no such square) *via* headphone and a new matrix appear. They are instructed to respond as fast and as correct as possible. Errors are either saying “yes” in the absence of a top-open square or saying no, when one was present. This task is a static visual search task. Figure 3 shows the visual task along with the stop task.

**The cognitive secondary task** is an auditory 1-back task. Numbers from zero to nine are read out loud *via* speakers in a random order and participants have to remember the penultimate number and repeat it verbally after the ultimate number. Participants are instructed to respond as fast and as correct as possible. Errors are either calling the wrong number or calling no number at all. This task is a memory task.

### Design and Dependent Measures

The experiment consists of a between-within mixed design with *road crossing task* and *secondary task* as within-subjects factors and *age group* as between-subjects factor. Both age groups perform the road crossing stop task and the road crossing go task as single tasks as well as with the visual secondary task and with the cognitive secondary task as dual-tasks.

Performance and subjectively perceived workload serve as dependent measures. Performance in the road crossing tasks and in the secondary tasks is operationalized as response time as well as errors and proportion of errors, respectively. While the two road crossing tasks were similar in with regard to the number of possible errors, the two secondary tasks’ structures were less similar to each other. This is why the comparison of error proportions is more suitable than the analysis of the total number of errors. Perceived workload is assessed with the NASA Task Load Index (NASA TLX; Hart and Staveland, 1988). This questionnaire allows the assessment of subjective workload *via* rating scales on the six dimensions: mental demand, physical demand, effort, performance, and frustration level.

### Procedure

Subjects participated in single subject sessions. On arrival, they read instructions and gave informed consent. Before the start of the experiment, a test of visual acuity (Landolt-Ring according to DIN EN ISO 8596) was performed. After training the visual and the cognitive task separately for 5 min each, baselines of performance in the secondary tasks were recorded. Then, they trained the two road crossing tasks. The experiment consisted of eight blocks. The first two blocks were single-task blocks of the two different road crossing tasks that served as baseline measures. Afterward, participants performed four dual-task blocks with all possible combinations of road crossing and secondary tasks in a counter balanced order. The last two blocks were again single-task baseline measures of the road crossing tasks. We decided to take two baselines, one in the beginning and one in the end to control for effects of learning and fatigue.

Each block had a duration of 5 min, containing 15 videos à 20s. After each of the experimental blocks (single and dual-task blocks), participants filled in the NASA TXL. In the end of the experiment, they answered the MoCA and a demographic questionnaire. Subsequently, they received financial compensation were thanked and dismissed.

The experiment had a total duration of approximately 3 h. Participants had three mandatory breaks of 5 min each and were allowed to take an additional break (between experimental blocks) whenever needed.

## Results

For comparison of road crossing task baselines to control for effects of learning and fatigue, three-way mixed ANOVAs were conducted with *road crossing task* (*stop* vs. *go*) and *block* (*first* vs. *last*) as within-subjects factors and age *group* (*older* vs. *younger*) as between-subjects factor.

To analyze performance in terms of errors and response time in the road crossing tasks, 2 (type of road crossing task) × 3 (dual-task condition: none, visual, cognitive) × 2 (age group) mixed AVOVAs were conducted. Performance in the secondary tasks was analyzed using 2 (type of secondary task) × 3 (dual-task condition: none, stop task, go task) × 2 (age group) mixed ANOVAs. The alpha level was set to 0.05 for all these analyses.

Comparison of the subjective workload (NASA-TLX) was done for the road crossing task and for the secondary task, using the same three-way within-between ANOVAs as for the performance measures.

Assumptions of sphericity were tested using the Mauchly test. In case of violation, Greenhouse–Geisser corrected values are reported.

### Comparison of Baseline Measures: Errors in the Road Crossing Tasks

The analysis of errors in the road crossing tasks revealed a significant main effect for block, *F*(1,36) = 5.36, *p* = 0.026, $\eta_{p}^{2}$ = 0.13. None of the other main effects and interactions was significant.

Overall participants made more errors in the first baseline block than in the last. This effect results form a decrease of errors made by the older group in the stop task (t1: *M* = 0.6; t2: *M* = 0.2) and a decrease of errors made by the younger group in the go task (t1: *M* = 0.5; t2: *M* = 0.3). The older groups’ performance in the go task (t1: *M* = 0.4; t2: *M* = 0.4) and the younger groups performance in the stop task (t1: *M* = 0.1; t2: *M* = 0.1) remained fairly stable over time.

We see a learning effect from the first to the second baseline measure, for both age groups, which is however, occurring in different tasks.

### Comparison of Baseline Measures: Response Time in the Road Crossing Tasks

The analysis of response time in the road crossing tasks revealed a significant main effect for the road crossing task *F*(1,36) = 85.19, *p* < 0.001, $\eta_{p}^{2}$ = 0.70, two significant interaction effects for—block × age group, *F*(1,36) = 16.34, *p* < 0.001, $\eta_{p}^{2}$ = 0.31, as well as for road crossing task × block, *F*(1,36) = 10.09, *p* < 0.001, $\eta_{p}^{2}$ = 0.18, and a significant triple interaction, road crossing task × block × age group, *F*(1,36) = 16.46, *p* = 0.003, $\eta_{p}^{2}$ = 0.22.

While response time in the stop task does not differ as a function of age or block (older: t1: *M* = 1.31 s; t2: *M* = 1.37 s; younger t1: *M* = 1.12 s; t2: *M* = 1.10 s), and older participants’ response time remains stable over time also in the go task (t1: *M* = 2.41 s; t2: *M* = 2.49 s), younger participants shorten their response time from the first to the second block in the go task (t1: *M* = 2.55 s; t2: *M* = 2.17 s). We find a learning effect with regard to response time only for the younger group for the go task.

The differences in errors and response time that we find for the two baseline blocks are not due to changes in the speed-accuracy trade-off but are learning effects. That can be said because the younger group in the go task is reducing both errors and response time and the older groups’ response time remains stable while reducing errors in the stop task. For further analyses, we will use the mean from the first and the second baseline block, as the first block is underestimating older participants’ performance in the stop task and younger participants’ performance in the go task.

### Performance in Road Crossing Tasks—Errors

The analysis of errors in the road crossing tasks revealed significant main effects for the type of road crossing task, *F*(1,36) = 8.43, *p* = 0.006, $\eta_{p}^{2}$ = 0.19, the dual-task condition, *F*(1.450,72) = 8.57, *p* = 0.002, $\eta_{p}^{2}$ = 0.19, as well as for the age group, *F*(1,36) = 95.42, *p* < 0.001, $\eta_{p}^{2}$ = 0.73. None of the interaction effects were significant.

**Figure 4:**
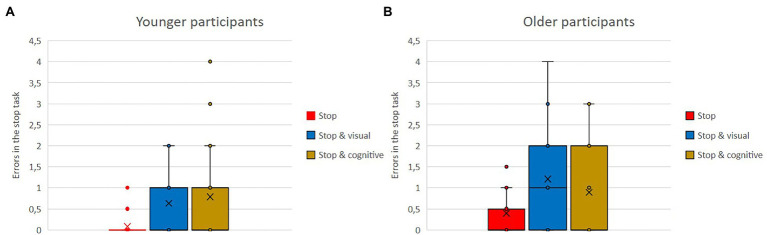
**(A,B)** Means of errors in the stop task, as single vs. dual-task conditions with either the visual or the cognitive secondary task for the older and the younger group.

**Figure 5:**
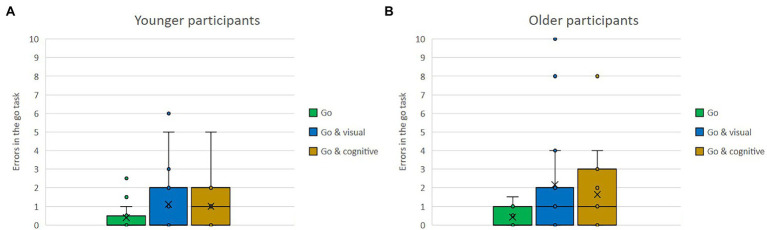
**(A,B)** Means of errors in the go task, as single vs. dual-task conditions with either the visual or the cognitive secondary task for the older and younger group.

Means of errors in the stop task and in the go task, as single versus dual-task conditions with either the visual or the cognitive secondary task for the older and the younger group, are shown in Figures 4A,B, 5A,B. Regardless of single versus dual-task conditions and age, overall participants made fewer errors in the stop task (*M* = 0.67) than in the go task (*M* = 1.12). Regardless of type of task and age, the number of errors was lower when tasks were conducted as single tasks than when conducted as part of a dual-task condition. That was true for the stop task (stop: *M* = 0.24 vs. stop and visual: *M* = 0.92, stop and cognitive: *M* = 0.84), as well as for the go task (go: *M* = 0.41 vs. go and visual: *M* = 1.63, go and cognitive: *M* = 1.32). Regardless of task type and conditions, overall, the younger participants made fewer errors (*M* = 0.67) than the older ones (*M* = 2.24).

### Performance in Road Crossing Tasks—Response Time

The analysis of response time in the road crossing tasks revealed significant main effects for the type of road crossing task, *F*(1,36) = 172.67, *p* < 0.001, $\eta_{p}^{2}$ = 0.83, the dual-task condition, *F*(2,72) = 14.43, *p* < 0.001, $\eta_{p}^{2}$ = 0.29, and the age group, *F*(1,36) = 6.44, *p* = 0.016, $\eta_{p}^{2}$ = 0.15. Two of the interaction effects reached significance, namely, type of road crossing task × dual-task condition, *F*(2,72) = 12.86, *p* < 0.001, $\eta_{p}^{2}$ = 0.26, and dual-task condition × age group, *F*(2,72) = 3.25, *p* = 0.044, $\eta_{p}^{2}$ = 0.08.

**Figure 6:**
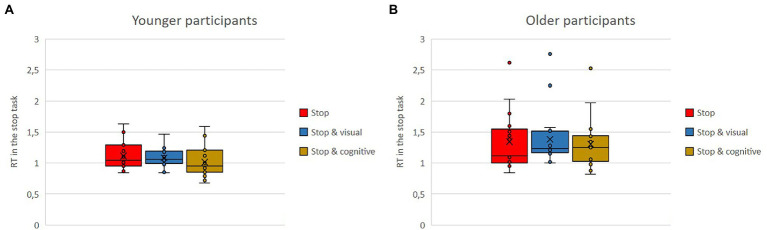
**(A,B)** Means of response time in seconds in the stop task, as single vs. dual-task conditions with either the visual or the cognitive secondary task for the older and the younger group.

**Figure 7:**
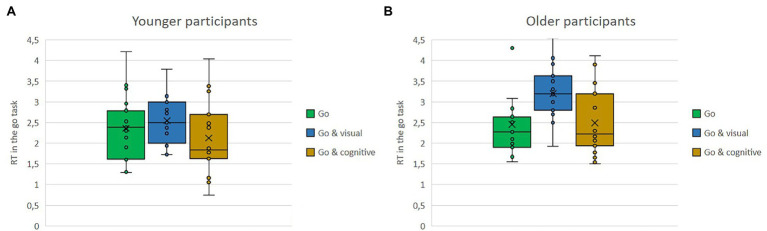
**(A,B)** Means of response time in seconds in the go task, as single vs. dual-task conditions with either the visual or the cognitive secondary task for the older and the younger group.

Means of response times in seconds in the stop task and in the go task as single versus dual-task conditions with either the visual or the cognitive secondary task for the older and the younger group are shown in Figures 6A,B, 7A,B. Regardless of single versus dual-task conditions and age, overall, participants’ response times were shorter in the stop task (*M* = 1.21) than in the go task (*M* = 2.53). Regardless of the type of task and the age, response times were shorter when the task was conducted in parallel to the cognitive secondary task (*M* = 1.73) compared to the visual secondary task (*M* = 2.22). Regardless of task type and conditions, overall, the younger participants had shorter reaction times (*M* = 1.71) than the older ones (*M* = 2.03).

The interaction effects are further qualified by the triple interaction. While in the stop task, there were no big differences in response time, whether being conducted as single or dual-task (stop: *M* = 1.23; stop and visual: *M* = 1.23; stop and cognitive: *M* = 1.16), and the same was true for the go task when conducted by the younger group (go single: *M* = 2.36; go and visual: *M* = 2.54; go and cognitive: *M* = 2.12), the older group was much slower when conducting the go task together with the visual task (go and visual: *M* = 3.20) compared to the other two conditions of go task alone or the combination of go task and cognitive task (go: *M* = 2.46, go and cognitive: *M* = 2.49).

### Performance in Secondary Tasks—Proportion of Errors

The analysis of proportion of errors in the secondary tasks revealed significant main effects for the type of secondary task, *F*(1,36) = 23.97, *p* < 0.001, $\eta_{p}^{2}$ = 0.40, the dual-task condition, *F*(2,72) = 5.39, *p* = 0.007, $\eta_{p}^{2}$ = 0.13, as well as for the age group, *F*(1,36) = 7.84, *p* = 0.008, $\eta_{p}^{2}$ = 0.18. One of the interaction effects reached significance, dual-task condition × age, *F*(2,72) = 3.61 *p* = 0.036, $\eta_{p}^{2}$ = 0.09.

**Figure 8:**
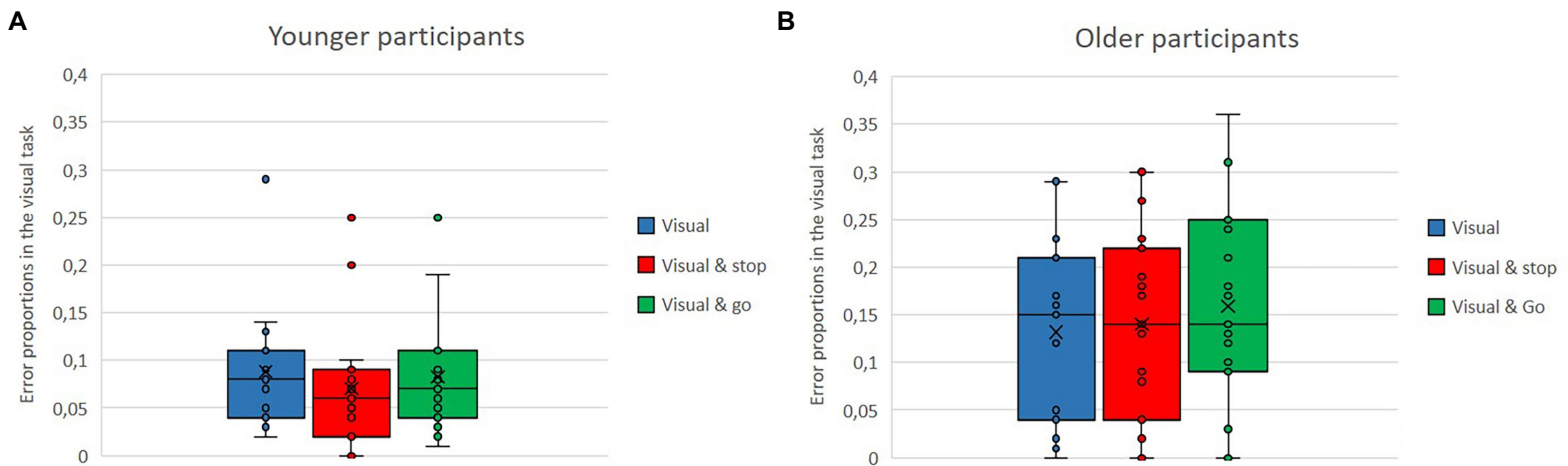
**(A,B)** Means of error proportion in the visual secondary task, as single vs. dual-task conditions either the stop or the go task for the older and the younger group.

**Figure 9:**
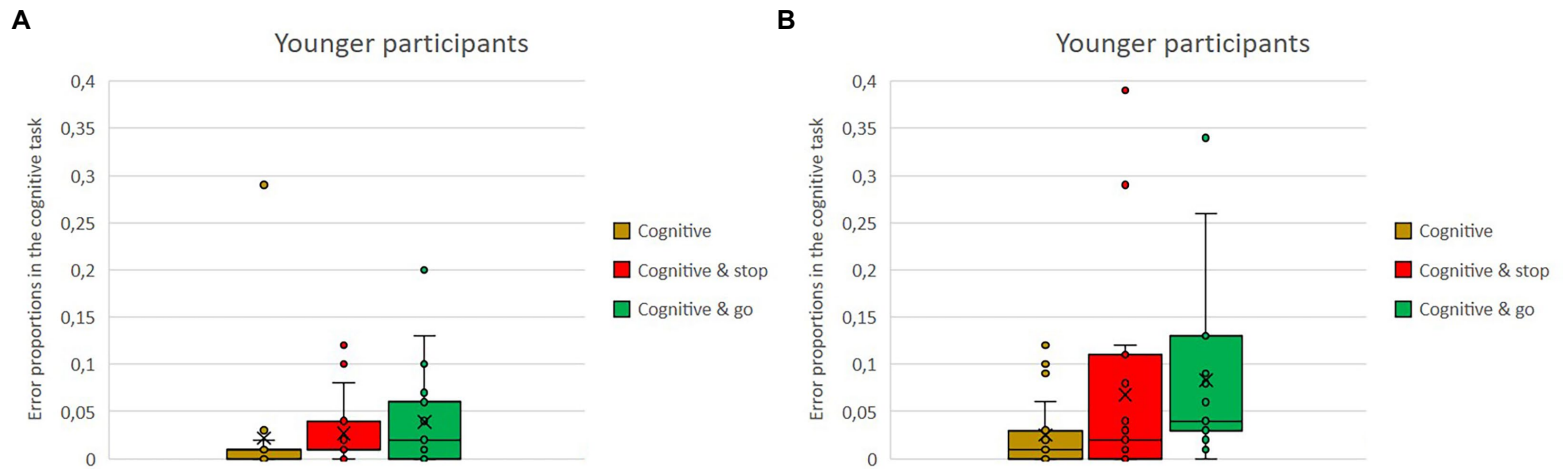
**(A,B)** Means of error proportion in the cognitive secondary task, as single vs. dual-task conditions with either the stop or the go task for the older and the younger group.

Means of error proportion in the visual secondary task and in the cognitive secondary task as single versus dual-task conditions with either the stop or the go task for the older and the younger group are shown in Figures 8A,B, 9A,B. Regardless of single vs. dual-task conditions and age, overall, participants made fewer errors in the cognitive task (*M* = 0.04) than in the visual task (*M* = 0.11). Regardless of task type and conditions, overall, the younger participants made fewer errors (*M* = 0.06) than did the older ones (*M* = 0.10). Overall, error proportion was lower in the single tasks than in the dual-task conditions (single: *M* = 0.07; dual: *M* = 0.08).

However, there is also the interaction of age and dual-task. While in the visual task there is only a difference depending on age, but error proportions in the single vs. dual-task conditions are rather similar within each group (younger: visual: *M* = 0.09, visual and stop: *M* = 0.07, visual and go: *M* = 0.08; older: visual: *M* = 0.13, visual and stop: *M* = 0.14, visual and go: *M* = 0.16), the pattern in the cognitive task is different. No age difference can be observed in the single-task condition (younger: *M* = 0.02, older: *M* = 0.03); however, there is a much higher error proportion in the dual-task conditions, but only for the older participants (younger: cognitive and stop: *M* = 0.03, cognitive and go: *M* = 0.04; older: cognitive and stop: *M* = 0.07, cognitive and go: *M* = 0.08).

### Performance in Secondary Tasks—Response Time

The analysis of response time in the secondary tasks revealed significant main effects for the type of secondary task, *F*(1,76) = 16.46, *p* < 0.001, $\eta_{p}^{2}$ = 0.18 and for the age group, *F*(1,76) = 16.46, *p* < 0.001, $\eta_{p}^{2}$ = 0.18. There was no main effect of dual-task condition, but an interaction effect of type of secondary task × dual-task condition, *F*(1,76) = 16.46, *p* < 0.001, $\eta_{p}^{2}$ = 0.18, and another interaction effect of type of secondary task × age group, *F*(1,76) = 16.46, *p* < 0.001, $\eta_{p}^{2}$ = 0.18.

**Figure 10:**
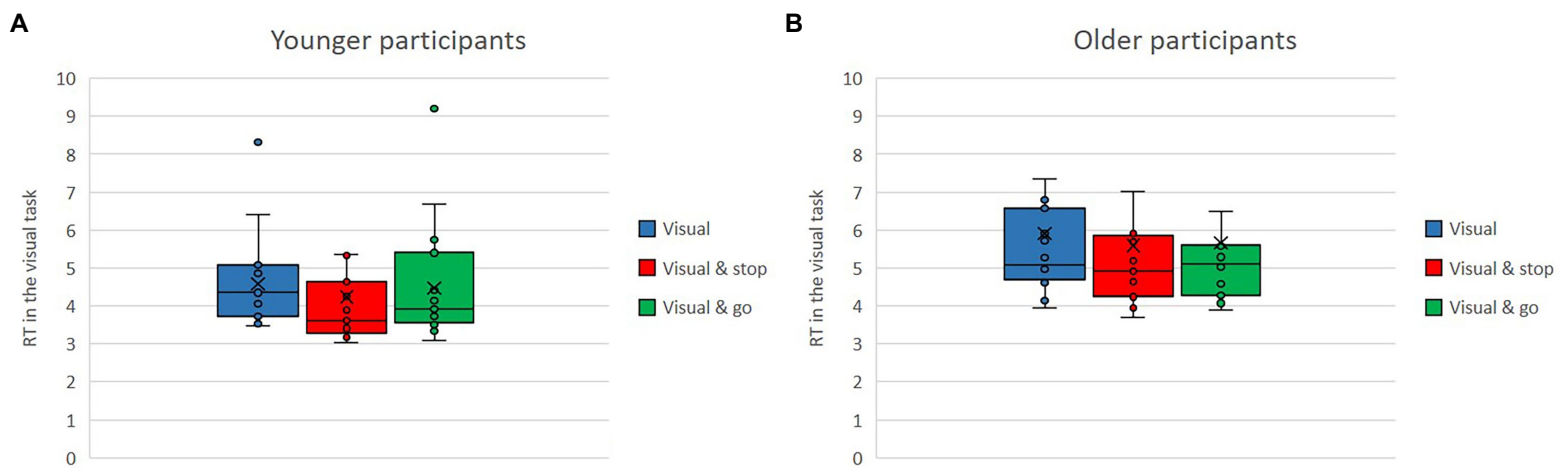
**(A,B)** Means of response time in the visual secondary task, as single vs. dual-task conditions with either the stop or the go task for the older and the younger group.

**Figure 11:**
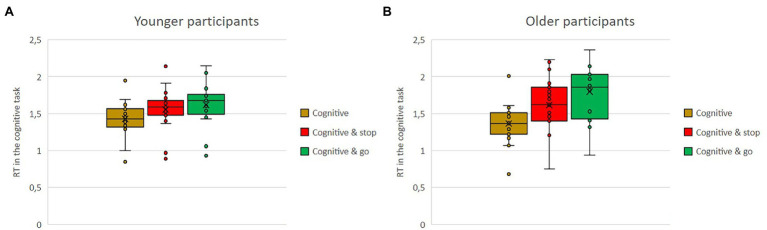
**(A,B)** Means of response time in the cognitive secondary task, as single vs. dual-task conditions with either the stop or the go task for the older and the younger group.

Means of response time in the visual secondary task and in the cognitive secondary task as single versus dual-task conditions with either the stop or the go task for the older and the younger group are shown in Figures 10A,B, 11A,B. Regardless of condition and age, response time was much shorter in the cognitive task (*M* = 1.56) compared to the visual task (*M* = 5.08). Regardless of task type and conditions, overall, the younger participants (*M* = 2.98) had a shorter response time than did the older ones (*M* = 3.66). The interaction between type of secondary task and dual-task task condition can be seen comparing Figures 10A,B, 11A,B. While response time in the cognitive task increased as a function of dual-task completion (single: *M* = 1.40, cognitive and stop: *M* = 1.58, cognitive and go: *M* = 1.7), responses in the visual task were even faster, when additional tasks were performed concurrently (single: *M* = 5.25, visual and stop: *M* = 4.92, visual and go: *M* = 5.07). The interaction between type of secondary task and age group manifests as significant age difference in response time in the visual condition (younger: *M* = 4.43, older: *M* = 5.72), compared to a rather similar performance for both age groups in the cognitive task is (younger: *M* = 1.54, older: *M* = 1.56).

### Subjective Workload (NASA) in Road Crossing Tasks

The analysis of subjective workload revealed a significant main effect of dual-task condition, *F*(2,72) = 103.62, *p* < 0.001, $\eta_{p}^{2}$ = 0.74. None of the other main effects and interaction effects was significant. Regardless of age and type of road crossing task, single-task completion led to fewer perceived workload (stop: *M* = 21.24, go: *M* = 20.57) than the dual-task completion (stop and visual *M* = 50.03; stop and cognitive *M* = 56.92; go and visual *M* = 51.24; go and cognitive *M* = 59.53).

### Subjective Workload (NASA) in Secondary Tasks

The analysis of subjective workload in secondary tasks revealed a significant main effect for dual-task condition, *F*(1.689,72) = 16.88, *p* < 0.001, $\eta_{p}^{2}$ = 0.32 and a significant interaction effect for type of secondary task × dual-task condition, *F*(1,76) = 6.94, *p* = 0.002, $\eta_{p}^{2}$ = 0.16. In the cognitive task, the workload is much lower in the single-task condition (*M* = 41.39) compared to the dual-task conditions (cognitive and stop: *M* = 56.92; cognitive and go: *M* = 59.53), whereas only very few differences can be found in the visual task between workload in the single-task (*M* = 45.58) versus the dual-task conditions (visual and stop: *M* = 50.03; *M* = 51.24).

## Discussion

With the help of the current experiment, we wanted to learn more about the impact of visual secondary task demands on the attention allocation of older pedestrians during the phase of traffic detection within the process of road crossing. For this purpose, we used two different road crossing tasks; one where an approaching car had to be detected (stop task) and another one where the resolution of a busy road situation had to be identified (go task). Two groups of participants (younger and older) completed the tasks as single versus dual-tasks, with either a visual or a cognitive secondary task. Performance was measured through errors and response time; in addition, subjective workload was assessed *via* NASA-TLX. Research questions regard the difference between the two age groups as well as the difference between the two road crossing tasks and the two secondary tasks.

### Performance in Road Crossing

With regard to errors, the stop task was easier for both age groups overall than the go task. This was true in the single-task condition as well as in the dual-task conditions.

Older participants made more errors in both road crossing tasks compared to younger participants. However, both age groups’ performance in the road crossing suffered proportionally as a function of dual-task condition. Thereby, the visual secondary task led to more errors than the cognitive secondary task. As there were no interaction effects, no age-specific dual-task cost could be identified for errors in the road crossing tasks.

When looking at the response times, we found participants to be faster in the stop task independent of age and single vs. dual-task condition. This finding is due to the different task characteristics (single detection vs. dynamic visual search) and thus, not surprising. However, when looking at age effects, the picture turns more complex. Older participants were slower in the stop task compared to the younger group. Both groups’ response times remained unchanged when a secondary task was introduced. In the go task, however, performance in the single-task condition was the same for both age groups. While it stayed fairly the same for the younger group, the older participants’ response time in the go task became slower, when they were additionally engaged in the visual secondary task. That was not the case with the cognitive task. Thus, based on the two interaction effects for road crossing task × other task and age × other task, we can identify an age-specific dual-task effect that manifests in slower responses to the traffic situation and takes place only in the combination of go task and visual task.

When comparing the errors and response time in the stop task descriptively, we found a preference of speed over accuracy, as both age groups kept their response times constant from single to dual-task condition, while accepting an increase in errors. In the go task, we saw a similar effect for the younger group for both secondary tasks and for the older group with regard to the cognitive task. Participants again kept the response time fairly stable, while making significant more errors. However, that was not the case for the older group when the visual task got added to the go task. This combination led to an increase of both errors as well as response time.

Independent of age, we found the strategy of maintaining the speed in traffic perception even when additional attentional demands were made. This behavior can be classified as unsafe, as it led to more errors in traffic perception regardless of age. However, as we know from former research (i.e., Avineri et al., 2012; Zito et al., 2015; Tapiro et al., 2016), older pedestrians face situations of additional visual demands (checking the ground for obstacles) much more often in real world scenarios than younger people. Thus, the more frequent overlooking of cars in older pedestrians (Statistisches Amt Berlin Brandenburg, 2020) can be explained through more frequent limited visual attention to traffic.

In addition, we see the age-specific dual-task costs in the combination of go task and visual task for response time and for errors. That means these two tasks have a strong interference. While the appearing car in the stop task triggers a certain pop out effect, the opposite is true in the go task condition. This task was classified as a dynamic visual search and includes a lot of distractors. As we know from basic research, peoples’ inhibitory control declines with advancing age (i.e., Tipper, 1991). Apparently, (healthy) older people are indeed perfectly able to understand when complex traffic situations resolve, but only when allocating the whole available amount of attention to this task of traffic perception. In case, an additional visual demand is added on top, as is the case in many crossing situations where older pedestrians feel the need to visually scan the ground for obstacles, traffic perception gets impaired. This finding gives another reason for older pedestrians’ lack of attention toward traffic that results in overlooking crossing cars.

### Performance in Secondary Tasks

Comparing performance in the visual and the cognitive task shows how different these tasks are. Participants’ performance in terms of error proportions and response time was independent of age better in the cognitive compared to the visual task.

In the visual task, younger participants performed better compared to older with regard to both error proportion and response time. Both age groups performance in the visual task did not suffer from dual-task effects. Error proportions and response time remained fairly stable during the different conditions with single versus dual-tasks.

The opposite picture emerges for the cognitive task. In the single-task condition, both groups’ performance did not differ. Error proportion and response time increased as a function of dual-task condition. While this is true for both groups, we found an over proportionally strong increase for the older group, which can be interpreted as age-specific dual-task cost. This effect is represented through the corresponding interaction effects.

Performance in the secondary tasks matches performance in the road crossing. In both road crossing tasks, performance decrements were stronger when the visual task instead of the cognitive task was conducted concurrently. In line with that, we see decrements in the cognitive task when conducted together with the road crossing, which is not the case for the visual task. Thus, it seems that attention allocation in the dual-task conditions differed based on the type of secondary task. When conducting the visual task, the secondary task was prioritized over the road crossing, which was not the case with the cognitive task.

This different strategy may be due to the nature of the two secondary tasks. While the cognitive task (as well as the road crossing tasks) can be classified as resource limited, the visual task seems to be rather data limited (Norman and Bobrow, 1975). The resource limited tasks on the one hand require resources that are apparently very limited and have great overlap, which often leads to the impairment of both concurrent tasks. That may be the very early working memory, which has to be proven amodal and thus, represents a bottleneck even though information from the road crossing task was visual and information from the cognitive task was auditory (Arnell, 2006). On the other hand, the visual task is data limited, and performance depends more on the quality of stimulus material and on the visual ability, which diminishes with age (i.e., Ball et al., 1990).

### Subjective Workload

Results of subjective workload correspond to the findings of dual-task effects. In both road crossing tasks as well as in the secondary task, the perceived workload increased as a function of dual-task condition, which is in line with performance measures that also decreased when a second task was added. In the visual task, performance did not differ between single versus dual-task conditions and nor did the perceived workload. For the other significant performance effects of age and type of tasks, no matching effects of subjective workload were found. It seems that NASA is not sensitive enough to mirror the behavioral effects on the level of subjective perception, much less able to gain information on further performance data.

### Theoretical Implications

When looking at all four tasks, it becomes inherent that there are two different types of tasks. On the one hand, the type of tasks that leads to age-specific dual-task effects, which manifest in interaction effects. Both age groups perform similar in the single task, but performance decreases due to dual-task demands are disproportionately stronger in the older group compared to the younger (Riby et al., 2004). These effects were found for performance in the go task and in the cognitive task, which both require cognitive resources in terms of working memory. Findings are in line with Salthouse’s general slowing hypothesis (e.g., Salthouse and Babcock, 1991; Salthouse, 1996). Accordingly, older peoples’ response time in each single cognitive process step is slowed down a little. When more steps are required, as it is the case in dual-task settings, the time loss accumulates and leads to a stronger decrease in overall response time and accuracy.

On the other hand, in the other type of tasks, we found age effects. Meaning older participants perform worse than younger, but when performance decreases from single to dual-task completion that age difference remains stable. This was the case in the stop task and in the visual task. Both tasks require mainly perceptual and less cognitive resources. With less cognitive processes involved, no accumulation of response time occurs. Differences between groups reveal age-related decrements of visual perception but remain stable in dual-task settings.

Thus, we can conclude that not only divided (visual) attention is an issue in road crossing dual-task scenarios. The interference of secondary tasks depends very much on the nature of these tasks as well as on the nature of road crossing tasks or even of the current phase of road crossing (traffic perception vs. traffic analysis and decision vs. actual crossing; Older and Grayson, 1974) or stage of road crossing task (e.g., perception vs. cognition vs. response; Wickens, 2002).

The real challenge is the understanding of the complexity of road crossing as well as the complexity of everyday tasks, such as talking at the phone (Neider et al., 2011), and linking it to age-related declines in the respective abilities, such as working memory (e.g., Salthouse, 1996). Deconstruction and separation of tasks as we have tried in this study can help to understand the separate mechanisms involved in handling complex traffic situations as an older pedestrian.

### Practical Implications

Due to consideration of internal validity, we chose artificial secondary tasks. While this limits the direct transfer to real world settings, it helps to better understand interference between different tasks. The visual task requires similar resources to the ones needed to scan the ground for obstacles. The cognitive task instead stands for all tasks conducted in parallel to road crossing that involve higher cognitive resources, such as demanding navigation, remembering shopping lists, or having an intense conversation.

Results of the current experiment help to broaden the horizon of knowledge regarding older pedestrians’ difficulties in attention allocation to traffic perception. The most important finding is the impaired performance in traffic perception through the parallel conduction of the visual search task, which was shown for both groups. Thus, every pedestrian scanning the ground for obstacles while looking for traffic risks misjudging the current traffic situation. In combination with the fact that older pedestrians are scanning the ground much more often than do younger ones (i.e., Wiczorek et al., 2016), this finding gives a valid reason for the more frequent car accidents of older compared to younger pedestrians.

Moreover, the impaired inhibition of dynamic distractors as a function of additional visual load was shown to be an age-specific problem. At first glance, this might not seem too relevant as car crashes would be associated with approaching cars rather than with cars driving away. However, a lot of real world traffic situations combine the two elements of hazard detection (stop task) and traffic resolution (go task), creating an even more complex and thus riskier situation. The reduced capacity of inhibition leads to an increased tendency to look at distractors (cars driving away), which in consequence enhances the risk to oversee targets (approaching cars). That means the possibility for car crashes with older pedestrians due to the overlooking of vehicles is even higher in complex (i.e., crowded) traffic situations.

Finally, it was found that dual-task situations with a visual task are more harmful to traffic perception than (auditory) cognitive tasks. However, also the cognitive task did reduce performance in road crossing. Thus, we can conclude traffic perception is a complex task that requires multiple resources (*cf*. MRM, Wickens, 2002) and can therefore be impaired by concurrent tasks of different modalities, focusing on different stages.

Of course one solution to the dual-task problem would be to perform tasks consecutively and not concurrently. This solution is easy in theory, but apparently difficult in practice. Public awareness campaigns could help to raise older pedestrians’ knowledge for this necessity.

Another practical implication for the public sector is the recommendation to repair as many surfaces as possible and maintain them in good shape. This reduces both older pedestrians’ risk of falling as well as their risk to get injured by a car.

## Data Availability Statement

The raw data supporting the conclusions of this article will be made available by the authors, without undue reservation.

## Ethics Statement

The studies involving human participants were reviewed and approved by Ethik-Kommission des Instituts für Psychologie und Arbeitswissenschaft (IPA) der TU Berlin. The patients/participants provided their written informed consent to participate in this study.

## Author Contributions

RW was mainly responsible for planning, conducting, and analyzing the experiment as well as for writing the article. JP assisted in planning and analyzing the experiment and assisted as well in writing the article. All authors contributed to the article and approved the submitted version.

## Funding

This research was funded by the Bundesministerium für Bildung und Forschung and the open access publication fee was funded by the Technische University Berlin.

## Conflict of Interest

The authors declare that the research was conducted in the absence of any commercial or financial relationships that could be construed as a potential conflict of interest.

## Publisher’s Note

All claims expressed in this article are solely those of the authors and do not necessarily represent those of their affiliated organizations, or those of the publisher, the editors and the reviewers. Any product that may be evaluated in this article, or claim that may be made by its manufacturer, is not guaranteed or endorsed by the publisher.
